# MRI-Derived Fetal Weight Estimation in the Midpregnancy Fetus: A Method Comparison Study

**DOI:** 10.1159/000519115

**Published:** 2021-11-17

**Authors:** Jacqueline Matthew, Emily Skelton, Lisa Story, Alice Davidson, Caroline L. Knight, Chandni Gupta, Dharmintra Pasupathy, Mary Rutherford

**Affiliations:** a School of Biomedical Engineering and Imaging Sciences and School of Life Course Sciences, Faculty of Life Sciences in Medicine, King’s College London, London, UK; b Guy’s & St. Thomas’ NHS Foundation Trust, London, UK; c North Tees and Hartlepool NHS Foundation Trust, London, UK; d Westmead Clinical School, Faculty of Medicine and Health, University of Sydney, Sydney, NSW, Australia

**Keywords:** Biometry, Fetal weight, Magnetic resonance imaging, Second trimester, Ultrasound, Volumetry, Preterm, Premature

## Abstract

**Objectives:**

The aim of this study was to compare the standard ultrasound (US) estimated fetal weight (EFW) and MRI volume-derived methods for the midtrimester fetus.

**Methods:**

Twenty-five paired US and MRI scans had the EFW calculated (gestational age [GA] range = 20–26 weeks). The intra- and interobserver variability of each method was assessed (2 operators/modality). A small sub-analysis was performed on 5 fetuses who were delivered preterm (mean GA 29 ^+3^ weeks) and compared to the actual birthweight.

**Results:**

Two MRI volumetry EFW formulae under-measured compared to US by −10.9% and −14.5% in the midpregnancy fetus (*p* < 0.001) but had excellent intra- and interobserver agreement (intraclass correlation coefficient = 0.998 and 0.993). In the preterm fetus, the mean relative difference (MRD) between the MRI volume-derived EFW (MRI-EFW) and actual expected birthweight (at the scan GA) was −13.7% (−159.0 g, 95% CI: −341.7 to 23.7 g) and −17.1% (−204.6 g, 95% CI: −380.4 to −28.8 g), for the 2 MRI formulae. The MRD was smaller for US at 5.3% (69.8 g, 95% CI: −34.3 to 173.9).

**Conclusions:**

MRI-EFW results should be interpreted with caution in midpregnancy. Despite excellent observer agreement with MRI volumetry, refinement of the EFW formula is needed in the second trimester, for the small and for the GA and preterm fetus to compensate for lower fetal densities.

## Introduction

Accurate estimated fetal weight (EFW) calculations are important to reliably screen for small for gestational age (SGA) fetuses and to detect and monitor fetal growth restriction (FGR) [1]. SGA typically refers to a fetus having an EFW or an abdominal circumference (AC) measurement of less than the 10th percentile for gestation or less than the 3rd percentile if severe [2]. FGR is not synonymous with SGA because some fetuses will be growth-restricted but achieve a normal birthweight; thus, FGR is considered a failure to achieve an “expected” growth potential [3, 4]. Nonetheless, fetuses that are correctly identified as severely SGA are significantly associated with FGR and poorer outcomes.

Early delivery at extreme prematurity is often considered only when the EFW is above 500 g [2, 5]. Thus, an accurate EFW in the second and early third trimester is crucial to ensure timely and appropriate interventions. Yet, the ultrasound (US) biometry-derived EFW (US-EFW) has significant random and inherent systematic variation (of up to ±15%) compared with birthweight, with errors more pronounced at the extremes of the normal range [6–9]. Recent studies suggest that a magnetic resonance imaging (MRI) whole fetal body volume-based EFW is far more reliable than the US-EFW at term, with errors compared to birthweight as low as 3% [10–18]. In 1994, Baker et al. [19] published the first MRI volume-derived EFW (MRI-EFW) formula, and it is the most widely used in the literature. Kacem et al. [14] proposed an alternative to the Baker formula and considered varying fetal density across the gestational age (GA) range. There is less evidence about the reliability of the MRI-EFW at a GA remote from term, when adverse health outcomes related to FGR can be more severe.

US operator training and audit is unlikely to improve measurement errors within US-EFW calculations significantly; thus, development of alternative methods is required [6]. 3D-US has shown some promise however has not been routinely adopted in the clinical setting because it is yet to be evidenced as being more reliable than 2D-US biometric methods [20–22]. The primary aim of this study was to compare the agreement and reliability of US and MRI to measure the EFW in the second trimester and a secondary aim to assess the feasibility of the MRI-EFW in cases of extreme prematurity.

## Materials and Methods

Healthy pregnant participants were prospectively recruited between November 2015 and April 2016 as part of the ethically approved intelligent fetal imaging and diagnosis (iFIND) project (ethics number: 14/LO/1806). Inclusion criteria were normal 20-week US anatomy scan, paired US and MRI data within 3 days of each other, and GA between 20 and 28 weeks.

For the secondary aim, participants at high-risk of preterm birth (PTB) were prospectively recruited between December 2015 and October 2017, for the “MRI quantification of fetal growth and development study” and as part of a subgroup described in 2020 by Story et al. [23] (ethics number 07/H0707/105). Inclusion criteria were GA 20–32 weeks, high-risk of PTB (i.e., asymptomatic women with either a history of previous PTB, late miscarriage [>16 weeks], or cervical surgery with a >50% risk of PTB in the next 2 weeks [calculated using a fetal fibronectin and cervical length algorithm]) [24, 25], and paired US and MRI data both within 10 days of delivery. Exclusion criteria for both control and high-risk PTB fetuses were known structural or chromosomal abnormalities, multiple pregnancies, inability to give informed consent, pregnancy complications such as pre-eclampsia or gestational diabetes, and contraindications to MRI such as claustrophobia or a recently sited metallic implant.

### Method Comparison of the EFW in the Second Trimester

The design for the primary aim was a prospective, blinded, within-subject paired method comparison, observer agreement, and reliability study at a single center. A Philips EpiQ US system (Philips Healthcare, Best, Netherlands) with a 6-1-MHz matrix probe was used to scan all control participants by 1 of 2 observers (J.M./C.K.) in a dedicated research US clinic. 2D-US anatomical image planes including the transventricular view of the fetal head for the head circumference (HC), transverse abdomen view for the AC, and long axis of the femur for the femur length (FL) measurement were identified during each examination and stored. Image plane selection criteria were obtained from the NHS Fetal Anomaly Screening Programme guidelines [26]. An image database containing anonymised US DICOM images was compiled using the Osirix image review software for offline measurement (version 7.5, Geneva, Switzerland). US databases were duplicated and randomised using a computer-generated randomiser before being reviewed offline for inter- and intraobserver variability by the 2 fetal imaging experts, blinded to previous imaging results and clinical history (including GA) as previously described [27]. Both US-observers used the first US database to independently measure 2D-US fetal biometry for interobserver measurements, and then US-observer 1 repeated the measures after a 6-week interval to generate intraobserver measurements.

The US-EFW was then calculated from the HC, AC, and FL biometry using the 3-parameter 1985 Hadlock formula [28]:

$$\text{US}-\text{EFW}(\text{g})=10^{{\left(1.326-0.00326\times \text{AC}\times \text{FL}+0.0107\times \text{HC}+0.0438\times \text{AC}+0.158\times \text{FL}\right)}^{4}}$$

**Fig. 1:**
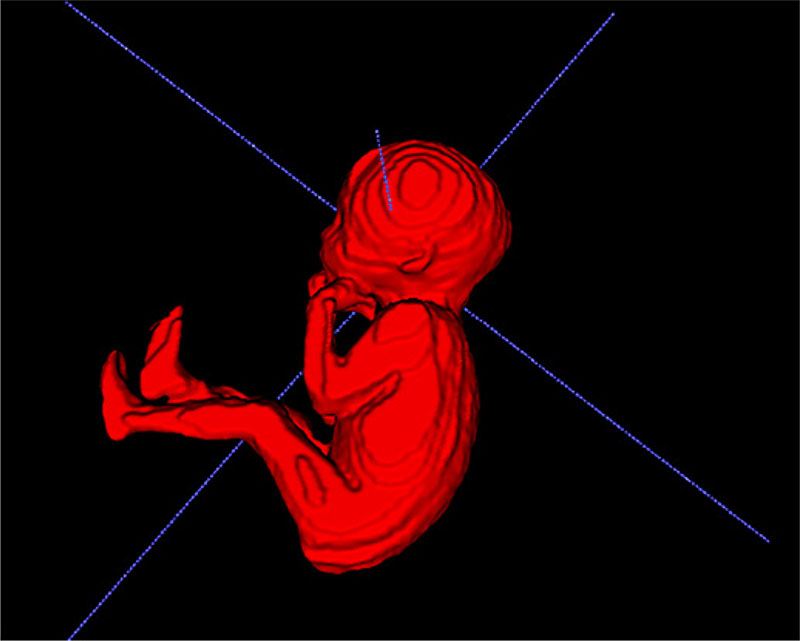
Semiautomatic segmentation of MRI whole fetal body volume.

The fetal MRI scan was performed using a Philips Ingenia 1.5 T MRI system (Philips Healthcare, Best, Netherlands). The mother was placed in a left lateral tilt, and no sedation was used for the examination. A sagittal plane orientated to the fetus was planned to acquire a balanced turbo field echo sequence which provided optimal image contrast resolution, coverage of the region of interest, and speed of acquisition (field of view = 420 × 420 mm; matrix = 288 × 288; repetition time = 4 ms; echo time = 1.98 ms; slices = 91; slice thickness = 5 mm; slice overlap = 2.5 mm; noise signal averages = 1/SENSE = yes [2]; flip angle = 90°; acquisition time = 1 min 25 s). MRI data from all the subjects were anonymized and randomized before being distributed to the fetal imaging experts (J.M./A.D.) for independent volume segmentation, blinded to US-EFW results and clinical history. Both MRI-observers calculated MRI whole fetal body volumes with a semiautomatic thresholding technique, and then performed manual slice-by-slice editing of the segmentation using open-source software, ITK-Snap (version 2.2.0),^33^ for interobserver measurements (see Fig. 1). All fetal body tissues were included in the segmentation even if some fetal tissues appeared misaligned between slices. Observer 1 (J.M.) performed repeated measures after a 6-week interval to generate intraobserver measurements.

The MRI-EFW was calculated using the 2 formulae below [14, 19]:

- Volume EFW_Baker_ (kg) = 1.031 × fetal body volume (L) + 0.12 (kg).
- Volume EFW_Kacem_ (kg) = 0.989 × fetal body volume (L) + 0.147 (kg).

All observers were provided with face-to-face training and given written guidance notes prior to the review explaining the required measurements, segmentation technique, and optimal viewing conditions for the review.

### Data Analysis

Data were recorded on an Excel spreadsheet (version 15.0, Microsoft Corp, Redmond, WA, USA) and analyzed using SPSS (version 26, SPSS Inc., Chicago, IL, USA). Statistical analysis was performed as per recommended guidelines to avoid study reporting variation [29–33]. For the primary aim, a power calculation determined that a sample size of 20 was required to give a power of 80% for a type 1 error of 5% to detect an effect size of 13.0 g difference (assuming a standard deviation of 104 g based on previous studies) [27, 34].

Normality testing was performed to ensure assumptions were met for statistical analysis. To assess the systematic differences between modalities, the mean difference in measurement from 2 observers per modality was compared with a 2-tailed paired *t* test. The average measures intraclass correlation coefficient (ICC) was used to test the intra- and interobserver agreement, with 95% confidence intervals. Predefined cutoff limits for the ICC were used: >0.99, very good; 0.95–0.99, good; 0.90–0.95, moderate; 0.70–0.90, poor; and <0.70, very poor [32]. Bland-Altman plots were used to graphically assess the mean difference in observations and their limits of agreement (LoA), and a linear regression coefficient was used to determine if there was a statistically significant proportional bias in the error as the fetal size increased. Finally, the proportion of cases which fell outside of 2 specified error thresholds (5% and 10%) was calculated.

### Preterm MRI-EFW Feasibility

Using the same MRI segmentation method, preterm fetuses meeting the inclusion criteria had the MRI-EFW calculated by a single observer (L.S.). The MRI-EFW of PTB cases were compared with the most recent clinical US-EFW and the actual birthweight (ABW) at delivery. Additionally, weight centiles were calculated from newborn and fetal population-based growth chart calculators [35, 36]. The ABW centiles were used to compute the expected EFW for each case and thus correct for any time interval between GA at delivery and GA at the time of the scan. The absolute and percentage differences between the expected and actual EFW were calculated for MRI and US.

## Results

### Observer and Subject Demographics

Twenty-five control cases met the inclusion criteria for the study’s primary aim. The mean maternal age at the time of the scan was 32.5 years (range 26–39). The mean maternal BMI was 26.3 kg/m^3^ (range 22.2–38.4 kg/m^3^). The mean gestational age at time of consent (i.e., the first scan) was 23^+4^ weeks (range 20^+2^–25^+5^ weeks). Seventeen participants (68%) underwent US and MRI on the same day, and 7 (32%) had the MRI scan exactly 3 days after US. Five cases did not have outcome data available as they delivered at a different center and were lost to follow-up. Of the 20 cases with outcome data, there was 1 preterm delivery at 31^+3^ weeks gestation which was included as a control because MRI and US examinations occurred within a short time interval. Including the preterm case, the median gestational age at delivery was 39^+4^ (range 31^+3^– 42^+0^ weeks), and the median birthweight was 3,240.0 g (range 1,850–4,480 g). For the method comparison, agreement, and reliability assessment, a total of 225 US images from 25 unique control subjects were measured by 2 US observers. This consisted of 75 images per database review, with US-observer 1 repeating the measures. A total of 75 MRI-observations from 25 unique MRI balanced turbo field echo sequences had a volume segmentation performed by 2 MRI observers in the same 25 control subjects, with MRI-observer 1 repeating the measures (see Table 1 for observer experience). For the secondary aim of the study, there were 5 high-risk PTB cases meeting the inclusion criteria for the feasibility aim of this study. The mean gestational age at the time of MRI was 29 ^+3^ weeks (range 25^+6^–31^+3^ weeks) and at US was 29^+1^ weeks (range 24^+6^–31^+3^ weeks). The mean gestational age at delivery was 29^+6^ weeks (range 26^+1^–31^+6^ weeks), and all were delivered within 9 days (median 5 days) of the US examination and 5 days (median 2 days) of the MRI scan. The mean ABW was 1,310 g, range 770–1,690 g. The 5 PTB cases had a clinical US examination performed by 3 different operators, and the MRI segmentation was performed by a single observer experienced in this method.

### Descriptive Statistics

**Fig. 2:**
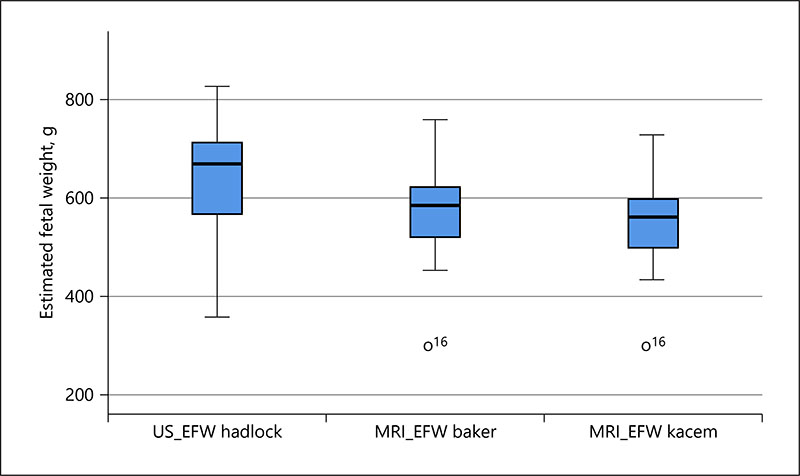
Boxplot of the EFW for health controls using US and MRI (Baker and Kacem) methods. EFW, estimated fetal weight; US, ultrasound; US-EFW, ultrasound biometry-derived estimated fetal weight; MRI-EFW, MRI volume-derived estimated fetal weight.

**Fig. 3:**
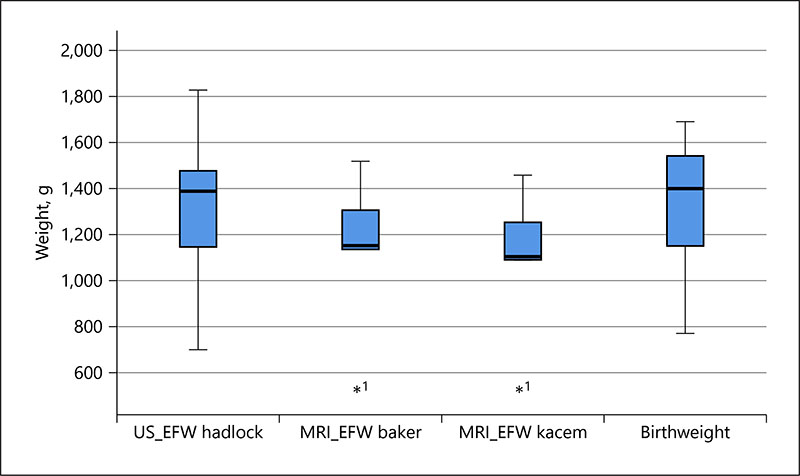
Boxplot of the EFW using US and MRI (Baker and Kacem) methods and the ABW for pregnancies resulting in preterm births. EFW, estimated fetal weight; US, ultrasound; ABW, actual birthweight; US-EFW, ultrasound biometry-derived estimated fetal weight; MRI-EFW, MRI volume-derived estimated fetal weight.

The MRI calculation using both formulae produce a smaller EFW in the same group of fetuses than the US-EFW, and the standard deviation is smaller for MRI than US, with the Kacem formula resulting in the smallest EFW and the least variation (see Fig. 2; Table 2). These differences demonstrate a similar pattern for the cases that delivered preterm (Fig. 3; Table 3).

### Differences between US and MRI for EFW (Healthy Controls)

Q-Q plots for normality testing demonstrated a linear relationship for EFW parameters, and a nonsignificant (<0.05) Shapiro-Wilk result was calculated. When compared to US-EFW, both MRI formulae consistently and significantly under-measure EFW. For the Baker method, the mean percentage error was −10.9% (70.7 g), and for the Kacem method, the mean percentage error was −14.5% (94.1 g). A significant difference between the 2 MRI-EFW methods was also demonstrated with a percentage error of −4.1% (23.5 g). All paired *t* test *p* values were significant and <0.001 (see Table 4).

### Inter- and Intraobserver Agreement

Excellent ICC scores were generated for intraobserver (0.998) and interobserver (0.993) MRI-EFW agreements. In comparison, the ICC score for the US-EFW was good for both intraobserver (0.972) and interobserver (0.984) agreements (see Table 5). The 95% confidence intervals overlap between US and MRI methods for both intra- and interobserver measurements, suggesting there is no significant difference in observer agreement between the modalities. The linear regression performed to assess proportional bias gave a statistical *p* value of >0.05 for every ICC result. This suggests that the agreement of US and MRI is independent of the overall size of the measurement taken at this GA range.

### Intra- and Interobserver Bland-Altman Plots of the US-EFW and MRI-EFW

The intraobserver mean relative (percentage) error of the MRI-EFW is lower than that of the US-EFW (0 and 4.5%, respectively) which indicates excellent MRI agreement, with US discordance being equivalent to 29.3 g which is statistically significant (see Table 6a). The interobserver relative mean percentage errors for the MRI-EFW and US-EFW are equally small (−1.8 and 1.1%, respectively), which represents an US mean absolute difference of −12.3 g to MRI’s 6.0 g, with the US difference reaching statistical significance. The *t* test for the direct comparison of US and MRI mean paired differences reached statistical significance, suggesting the observer variation between the modalities when measuring the EFW is real (see Table 6b).

**Fig. 4:**
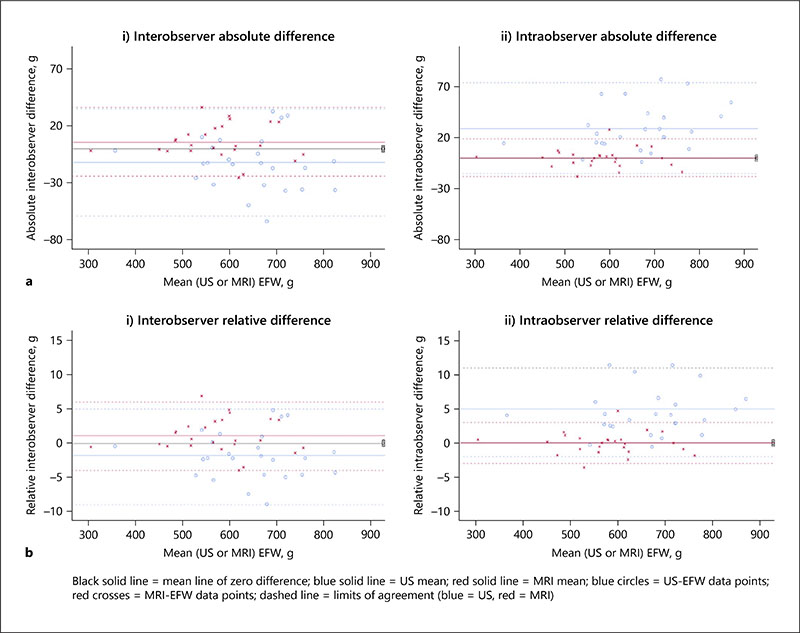
Bland-Altman plots of the absolute (**a**) and relative (**b**) differences for interobserver (i) and intraobserver (ii) EFW measures. EFW, estimated fetal weight; LoA, limits of agreement.

Bland-Altman plots graphically represent the absolute and relative mean difference and the LoA, that is, the variation in 95% of the dataset (or ±1.96 SD from the mean) for each US-EFW and MRI-EFW datapoint (see Fig. 4). US-EFW LoA is wider than that for the MRI-EFW for both intra- and interobservations, suggesting MRI is more precise. For interobserver MRI-EFW measures, 95% of the cases are within ±29.8 g, and for US-EFW, they are within ±44.8 g (see Table 6a). EFW data-points for both MRI and US appear randomly spread on the charts, which suggests no proportional bias and confirms the findings of the linear regression statistics (Table 5).

### Absolute Error Thresholds

Threshold values for an arbitrary, but clinically relevant, cut off in variability (random error) for the EFW of 10% and then 5% assess the proportion of cases which fell outside these ranges compared to the mean of the intraobserver repeated measures which are usually the smallest in error (Table 7). For the US-EFW, there were 2 out of 25 cases (8%) for intraobserver calculations, but no interobserver cases that fell outside of the 10% error threshold. However, there were 8/25 cases (32%) and 5/25 (20%) for intra- and interobserver calculations, respectively, that fell outside of the 5% threshold. For the MRI-EFW, there was only 1 case that had an error of >5%. There were no other cases falling outside the error threshold for the MRI-EFW, confirming the precision of MRI over US.

### Preterm Sub-Analysis

**Fig. 5:**
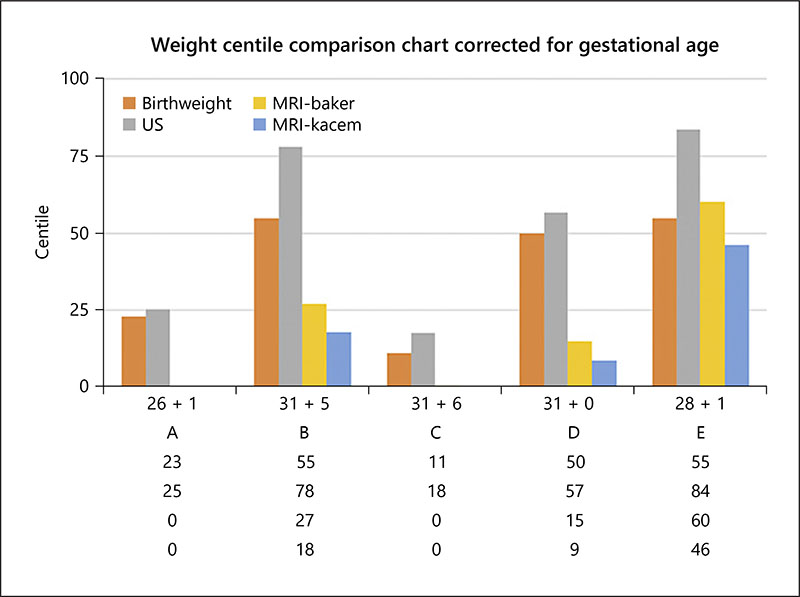
Actual US and MRI-EFW centiles, corrected for GA at the time of scan, compared to the birthweight centile. EFW, estimated fetal weight; US, ultrasound; GA, gestational age.

For the 5 preterm fetuses, all were delivered within 5 days of the MRI examination, and when the expected estimated weight was calculated using the ABW centiles and thus controlling for GA, there was a clinically significant mean difference between the actual and expected MRI-EFW of −13.7%/−159.0 g (Baker) and −17.1%/−204.6 g (Kacem) (see Tables 3, 8). In contrast to the MRI-EFW, the US-EFW overmeasured birthweight compared with the expected EFW by a mean of 5.3%/69.8 g. In 1 case, the MRI-EFW demonstrated a smaller relative difference than US, 1.5%–10.1%, respectively (case E). However, 4 of 5 MRI cases had a relative difference from the expected EFW of >5%. For the US 2 cases, cases B and E had a relative difference of >5%. When comparing the weight centiles, corrected for GA at the time of scan, US appeared to overmeasure consistently compare to the ABW centile and MRI appeared to under-measure consistently, except in case E, where the MRI estimate was accurate (see Fig. 5).

## Discussion

Our study showed higher observer reliability for MRI-EFW calculations than for US-EFW calculations, suggesting better reproducibility, repeatability, and precision of the MRI method in the second trimester. Yet, caution must be exercised if using this technique for the EFW in small fetuses as the MRI calculation showed systematic measurement differences compared to US on which fetal growth trajectory charts are based.

A recent literature search revealed no studies focusing on volumetric MRI-EFW for mid-second trimester fetuses using either the Baker or the Kacem formula and then comparing the results to US and/or birthweight. However, a few studies have looked at 2D-MRI biometry to estimate fetal weight in the second trimester with limited success [27, 37]. Our results contrast with recent findings by Kadji et al. [10] who assessed observer variability in EFW calculation for MRI and US in full-term fetuses. In their study, the mean relative error in the EFW difference for MRI and US was 0.9% and −0.8%, respectively, for intraobserver measures (0.0% and −4.5% in our study) and 0.6% and 0.5% for interobserver measurements (1.1% and −1.8% in our study). Effectively, Kadji et al. [10] suggest excellent agreement for both modalities; however, our study agreement suggests excellent MRI agreement but with US being slightly less precise within and between observers. Other studies report that a significant proportion of US random errors (between 58 and 80%) is incurred through observer variations in caliper placement, and for these cases, training and quality audit will help to some degree but not entirely [14, 38–40].

Kadji et al. [41] found a random error of ±1.9% and 8.8% in MRI and US, respectively, for intraobserver measures (±1.6% and 3.4% in our study) and ±2.8% and 11.2% for interobserver measurements (±2.6% and 3.5% in our study). The larger US errors reported by Kadji et al. [41] are likely to reflect increased proportional bias, observed when random errors increase as US measurements become larger at a later GA (in this case, term), a phenomenon described by obstetric US-observer variation studies [38]. In our sample (second trimester), this high proportional variation is not seen with US due to smaller fetal sizes. Nonetheless, the MRI-EFW is less susceptible to higher proportional variation likely because of the use of one volumetric parameter with well-defined landmarks rather than linear measurements subject to the US caliper-placement error. In our study, as in the Kadji study, regression analyses found no statistically significant difference in EFW variation as a result of increasing GA; however, these findings are based on narrow GA ranges under observation [29].

The PREMACRO study [41] found that for term fetuses, the mean relative errors of MRI were between 2.6– 3.7% and 6.3–11.4% for the US-EFW, compared to birthweight when calculated <1 week of delivery. Other studies also found small relative differences (between 3 and 4%) for MRI volume weight estimates using the Baker formula when compared to the weight at term [14, 15, 19, 42]. Our preterm sub-analysis compared the actual MRI-EFW to the expected EFW based on the ABW centile, and the mean relative error was larger and clinically significant for the MRI-EFW than the US-EFW (−14 to −17% and 5%, respectively). While larger studies would be required to investigate these contrasting findings further, it is an important study because it suggests that while the MRI-EFW may have less observer variation, the MRI-EFW calculation demonstrates systematic under-measuring of the fetal weight at GAs remote from term when compared to the US estimated and ABW, whereas the literature suggests very good MRI performance >37 weeks.

The Kacem study found the proportion of cases that fell outside the ±10% random error threshold was 26.6% for US cases and 1.1% for MRI, when the EFW was compared to birthweight (20% and 4% for US and MRI interobserver variation with stricter 5% threshold in our study) [14]. In 2003, Zarestsky et al. [15] found 15% of US and 5% of MRI cases fell outside the ABW ±10% threshold, and when using a ±5% error threshold, 73% of US cases and 49% of MRI cases fell outside the threshold [15].

The segmentation process in our study used a manual and semiautomated method; however, more recently automated planimetry techniques have been described to produce the MRI fetal body volume [43]. Although small errors were seen for the MRI-EFW, the segmentation technique and choice of acquisition sequence may have an impact on the differences between studies. In addition, varying fetal tissue density (fat, bone, and muscle) at different gestations may be responsible for the MRI-EFW formula not performing well remotely from term [16]. Kacem used a linear regression model to attempt to address this issue, modifying the original Baker formula, although only 24 of 188 cases were <37 weeks GA, and this could have resulted in poorer generalizability of their EFW formula at low gestations. Fetal MRI studies, where fetal fat volumes have been reliably measured in utero, confirm varying fat densities across GAs with negligible fat deposits at around 28 weeks [44]. In the future, MRI may further aid our understanding of developmental fat depositions and lead to more accurate EFW formulae in the setting of FGR or gestational diabetes [45, 46].

### Role of MRI

As MRI acquisition speed and affordability improves, MRI will become more important for the preterm fetus, when US quality is limited and an accurate EFW is clinically relevant. Although yet to be fully established in the care pathway, clinical validation of MRI applications must be emphasised, particularly as new MRI-specific fetal growth charts and MRI organ volumes indexed to whole fetal volume are developed [47, 48].

While this study provides needed insight into how the MRI-EFW performs at GA remote from term, there are some limitations. The semiautomated MRI segmentation technique used in this study is currently time-consuming (approximately 30 min/case). Other authors have described bespoke planimetry and manufacturer-based automated segmentations which can further increase reliability and reduce the postprocessing time to 5 min [49, 43]. Future work should address the development of MRI 3D motion-corrected tools for the fetal body and fully automated techniques for volume extraction. This could include an assessment of methods to address artifacts from reconstructions or segmentation techniques, for example, slice interpolation or 3D smoothing algorithms [50, 51].

The primary comparator was US as the gold standard; however, US is subject to well-documented observer subjectivity and measurement error. In the PTB sub-analysis, the MRI-EFW and US-EFW comparator was derived from the ABW; however, charts to calculate birthweight centiles are based on different populations to the fetal growth charts and also takes into account fetal sex [52, 53]. Weight at birth is likely to be physiologically larger to weight in utero due to the mode of delivery, whereby cesarean newborns typically weigh more than standard vaginal deliveries, and the use of intravenous fluids during labor has been seen to artificially increase newborn weight as a result of this intervention [54–56]. These factors were not controlled for in this study, although clinically appropriate newborn and fetal growth charts and use of centiles helped to standardize the comparisons.

## Conclusion

The US-EFW remains the preferred method for fetal growth assessment. Clinicians must be aware of the limitations due to the measurement error and potential clinical implications of using the US-EFW to inform patient management. The MRI-EFW has far lower observer variation than that of US, but the current formulae (Baker and Kacem) are not reliable for the midpregnancy or extremely preterm fetus.

## Figures and Tables

**Table 1:** Operator experience

	Role	Fetal imaging experience, years
US-observer 1	Reporting obstetric sonographer	3
US-observer 2	Fetal medicine consultant	6
MRI-observer 1	Reporting obstetric Sonographer/MRI radiographer	13
MRI-observer 2	Fetal MRI data analyst/researcher	5
Preterm Birth MRI observer	Fetal medicine sub-specialty trainee/clinical academic	5

**Table 2:** Mean US-EFW (Hadlock formula) and MRI-EFW (Baker and Kacem formulae) for 25 midpregnancy fetuses in grams (SD)

	Observer 1	Observer 2	Observer 1 (repeated measure)	Mean (observer 1 and observer 2)
Mean US-EFW, Hadlock	653.3 (106.4)	641.0 (103.6)	682.6 (113.7)	647.1 (104.2)
Mean MRI-EFW, Baker	579.5 (99.3)	573.5 (99.6)	579.7 (99.8)	576.5 (99.1)
Mean MRI-EFW, Kacem	556.1 (95.8)	550.1 (95.5)	555.9 (95.3)	553.0 (95.1)

SD, standard deviation; US-EFW, ultrasound biometry-derived estimated fetal weight; MRI-EFW, MRI volume-derived estimated fetal weight.

**Table 3:** High-risk preterm birth results for ABW and expected and actual EFW for US and MRI

Case	GA delivery	US to delivery interval, days	MRI to delivery interval, days	ABW, g	ABW centile^$^	Expected US-EFW, g^*^	Actual US-EFW, g	Expected MRI-EFW, g^*^	Actual MRI-EFW_Baker_, g	Actual MRI-EFW_Kacem_, g
A	26 + 1	9	2	770	23rd	664	700	745	534	513
B	31 + 5	2	2	1,690	55th	1,637	1,827	1,637	1,518	1,456
C	31 + 6	6	5	1,400	11th	1,284	1,389	1,302	1,136	1,090
D	31 + 0	5	3	1,540	50th	1,419	1,476	1,466	1,306	1,253
E	28 + 1	5	0	1,150	55th	1,052	1,144	1,150	1,150	1,104
Mean	29 + 3	5	2	1,310	39th	1,211	1,307	1,260	1,129	1,083
SD	(Range 26 + 1 to 31 + 6)	2.2	1.6	323.3	20.5	333.0	374.4	304.5	327.8	314.1

ABW, actual birthweight; EFW, estimated fetal weight; US-EFW, ultrasound biometry-derived estimated fetal weight; MRI-EFW, MRI volume-derived estimated fetal weight; US, ultrasound; GA, gestational age. g, grams.

$ Centile calculated using WHO-UK chart [35].

* Expected weight calculated using $ and Intergrowth fetal charts [36].

**Table 4:** Paired *t* test of the differences between the mean US-EFW and MRI-EFW (Baker and Kacem)

*N = 25*	Mean US-EFW, g	Mean MRI-EFW, g	Absolute mean paired difference, g (95% confidence interval)	Relative mean paired difference, %	*p* value
US versus MRI Baker EFW	647.1	576.5	70.7 (50.1–91.2)	−10.9	<0.001
US versus MRI Kacem EFW	647.1	553.0	94.1 (73.8–114.5)	−14.5	<0.001
	Mean MRI Baker EFW, g	Mean MRI Kacem EFW, g	Absolute mean paired difference, g (95% confidence interval)	Relative mean paired difference, %	p value
MRI Baker versus MRI Kacem EFW	576.5	553.0	23.5 (21.8–25.1)	−4.1%	<0.001

US-EFW, ultrasound biometry-derived estimated fetal weight; MRI-EFW, MRI volume-derived estimated fetal weight; US, ultrasound.

**Table 5:** Intra- and interobserver agreement, ICC (95% confidence intervals), linear regression *p* value for proportional bias

	US-EFW	MRI-EFW
Intraobserver (within observer)	**0.972, good** (0.558–0.993) *p* = 0.268	**0.998, excellent** (0.989–0.998) *p* = 0.874
Interobserver (between observers)	**0.984, good** (0.956–0.993) *p* = 0.254	**0.993, excellent** (0.984–0.997) *p* = 0.659

ICC, intraclass correlation coefficient; US-EFW, ultrasound biometry-derived estimated fetal weight; MRI-EFW, MRI volume-derived estimated fetal weight.

**Table 6:** (a) Absolute and relative inter- and intraobserver mean difference, with single measures *t* test and (b) absolute and relative inter- and intraobserver mean paired difference between US and MRI, with a paired *t* test

	Modality*	Mean difference	SD (±1.96 SD)	Sig. (2-Tailed)
Interobserver absolute difference, g	US	−12.3	23.9 (46.8)	*0.017*
	MRI	6.0	15.2 (29.8)	0.059
Interobserver relative difference, %	US	−1.8	3.5 (6.9)	*0.016*
	MRI	1.1	2.6 (5.1)	*0.043*
Intraobserver absolute difference, g	US	29.3	22.6 (44.3)	*<0.001*
	MRI	−0.2	9.5 (18.6)	0.898
Intraobserver relative difference, %	US	4.5	3.4 (6.7)	*<0.001*
	MRI	0.0	1.6 (3.1)	0.891
Absolute interobserver US and MRI paired difference, g		−18.3	31.1 (61.0)	*0.007*
Relative interobserver US and MRI paired difference, %		−2.9	4.7 (9.2)	*0.005*
Absolute intraobserver US and MRI paired difference, g		29.6	25.2 (49.4)	*<0.001*
Relative intraobserver paired difference, %		4.5	3.8 (7.4)	*<0.001*

g, grams; %, percentage; SD, standard deviation; sig., significance, presented as a *p* value (italics = *p* value <0.05). US, ultrasound; EFW, estimated fetal weight.

* As both MRI formulae used the same fetal volume measurement, their relative variation (random error) will be the same; therefore, the Kacem generated EFW is not presented.

**Table 7:** Proportion of US-EFW and MRI-EFW cases falling outside of 10% and 5% arbitrary error threshold

Arbitrary cutoff percentage	Threshold value, grams, g	Intraobserver		Interobserver	
		US-EFW *n* (%)	MRI-EFW*, *n* (%)	US-EFW, *n* (%)	MRI-EFW*, *n* (%)
US-EFW 10%	66.8	2 (8)		0 (0)	
US-EFW 5%	33.4	8 (32)		5 (20)	
MRI-EFW 10%	58.0		0 (0)		0 (0)
MRI-EFW 5%	29.0		0 (0)		1 (4)

EFW, estimated fetal weight; US-EFW, ultrasound biometry-derived estimated fetal weight; MRI-EFW, MRI volume-derived estimated fetal weight.

* As both MRI formulae used the same fetal volume measurement, their random error will be the same; therefore, the Kacem generated EFW is not presented.

**Table 8:** High-risk PTB absolute and relative differences between expected and actual EFW for US and MRI

Case	US absolute difference, g	US relative difference, %	MRI Baker absolute difference, g	MRI Baker relative difference, %	MRI Kacem absolute difference, g	MRI Kacem relative difference, %
A	5.0	0.7	−249.0	−31.8	−270.0	−34.5
B	152.0	9.1	−157.0	−9.4	−219.0	−13.1
C	54.0	4.0	−219.0	−16.2	−265.0	−19.6
D	31.0	2.1	−187.0	−12.5	−240.0	−16.1
E	107.0	10.3	17.0	1.5	−29.0	−2.6
Mean difference, SD (95% CI)	69.8, 53.1 (−34.3 to 173.9)	5.3, 3.8 (−2.2 to 12.7)	−159.0, 93.2 (−341.7 to 23.7)	−13.7, 10.8 (−34.9 to 7.5)	−204.6, 89.7(−380.4 to −28.8)	−17.1, 10.4 (−37.5 to 3.2)

EFW, estimated fetal weight; PTB, preterm birth; US, ultrasound.

## Data Availability

The collected and analysed data sets used during the current study are available from the corresponding author upon request. Publication and distribution of MRI iFIND project data (14/ LO/1086) are currently ongoing.
